# Anti-NF155/NF186 IgG4 Antibody Positive Autoimmune Nodopathy

**DOI:** 10.3390/brainsci12111587

**Published:** 2022-11-20

**Authors:** Lijun Wang, Jing Pan, Huanyu Meng, Zhao Yang, Lili Zeng, Jun Liu

**Affiliations:** 1 Department of Neurology and Institute of Neurology, Ruijin Hospital, Shanghai Jiao Tong University School of Medicine, Shanghai 200025, China; 2 Department of Neurovascular Center, Changhai Hospital, Naval Medical University, Shanghai 200433, China; 3 Department of Neurology, Ruijin North Hospital, Shanghai Jiao Tong University School of Medicine, Shanghai 200025, China; 4 CAS Center for Excellence in Brain Science and Intelligence Technology, Ruijin Hospital, Shanghai Jiao Tong University School of Medicine, Shanghai 200025, China

**Keywords:** autoimmune nodopathy, neurofascin 155, neurofascin 186, IgG4, rituximab

## Abstract

Patients with chronic inflammatory demyelinating polyradiculoneuropathy (CIDP) seropositive for autoantibodies against nodal and paranodal proteins display distinct clinical presentations. In the latest study, CIDP with autoantibodies against paranodal proteins was defined as autoimmune nodopathy (AN). We herein present a case of 39-year-old male with anti- neurofascin (NF) 155 and NF186 IgG4 antibody with gait disturbance and tremor, who was followed up for 4 months and demonstrated clinical improvements after apparently effective rituximab therapy. In addition, a literature review was conducted to investigate the clinical characteristics of anti-NF155/NF186-positive AN.

## 1. Introduction

In recent years, some chronic inflammatory demyelinating polyradiculoneuropathy (CIDP) specific biomarkers have been discovered successively, including antibodies such as anti-neurofascin (NF)155 [1,2,3,4,5], anti-contactin-1 (CNTN1) [6,7,8], anti-NF186 [5,9,10,11], and anti-contactin-associated protein 1 (CASPR1) [12]. Each autoantibody-related CIDP subtype has its own clinical features. Among these nodal-paranodal antibodies, are predominantly of the IgG4 subtype [1]. In the latest update of the European Academy of Neurology/Peripheral Nerve Society CIDP diagnostic guideline, considering patients with these antibodies often have specific clinical characteristic features and pathological manifestations, CIDP with autoantibodies against paranodal proteins are referred to as autoimmune nodopathies (AN) [13].

Anti-NF155 IgG4 antibody had been found in patients with AN, these patients were associated with younger age of onset, higher frequency of drop foot, gait disturbance, sensory ataxia, tremor, distal acquired demyelinating symmetric (DADS) neuropathy phenotype, higher cerebrospinal fluid (CSF) protein levels, and nerve conduction studies (NCS) showed that the distal and F-wave latencies were more significantly prolonged, as well as the effects of intravenous immunoglobulin (IVIG) was poor [1,2,4,14,15,16,17,18].

Meanwhile, antibodies against NF186 were found in less than 3% of patients with AN [5,9,10,11,18,19,20,21]. Delmont et al. reported that anti-NF186 IgG was related to a subset of AN patients with subacute-onset, sensory ataxia, demyelination with conduction block, cranial nerve involvement and concomitant autoimmune disorders. No patient showed tremor or neuropathic pain.

Here, we firstly report the presence with nodo/paranodal protein NF155/NF186 IgG4 antibodies in a Chinese patient with AN and summarize the clinical characteristics, electrophysiological, pathological description and following therapy.

### Case Presentation

A 39-year-old man presented with an 18-month history of numbness of limbs and 17-month distal weakness of lower limbs to our hospital in May, 2021. In October 2019, he felt numbness at the end of the fingers and toes, and more severe in the lower limbs. After one month, the numbness gradually developed to the bilateral limbs and appeared the lower extremity weakness. So, he went to the local hospital in November 2019. The nerve conduction velocity (NCV) showed that the conduction function of the common peroneal nerve and tibial nerve was decreased. CSF indicated albuminocytologic dissociation. He was diagnosed as Guillain-Barre syndrome (GBS). But the patient refused IVIG treatment for economic factor. Finally, he received steroid pulse therapy and the symptoms were improved after the treatment. In January 2020, he appeared to gait instability, accompanied with a feeling of stepping on cotton. In March 2020, He found bilateral lower limbs began to become thinner, and he went to the local hospital to receive IVIG treatment, but the symptoms were not ameliorated. In April 2020, he went to the Ruijin Hospital Luwan branch, the CSF showed albuminocytologic dissociation, Pandy’s test was strong positive (+++), protein was 2447 mg/L, the leukocyte count was 1.3 × 10^6^/L, and he was diagnosed as CIDP and treated with 4.3 g of methylprednisolone (500 mg × 5 days + 240 mg × 5 days + 120 mg × 5 days). After treatment, the numbness of limbs improved better than before. On 22 May 2020, He went to Ruijin Hospital Luwan branch and received 800 mg cyclophosphamide pulse therapy. After that, the patient received cyclophosphamide 400–800 mg pulse therapy every 2 months (total 4.8 g). He appeared bilateral upper limb’s tremor in October 2020. Since onset, he has normal urination and defecation, and no significant changes in body weight.

He denied family history, past history and personal history. There was no additional history of fever, trauma, infection, or vaccination. There was no obvious abnormality in the physical examination of the internal medicine system.

Neurological examinations showed low frequency tremor at both arms, ataxic-stepping gait, the muscle strength of bilateral upper limbs was grade V, the muscle strength of bilateral lower limbs was grade V-, bilateral lower limbs tendon reflexes were diminished, muscle atrophy could be seen in bilateral lower limbs, with high arch feet, deep sensory impairment, and decreased acupuncture sensation on both sides of the ankle joints below 2–3 transverse fingers. Bilateral finger-nose test was negative, bilateral heel-knee-shin test was positive, Romberg’s sign was positive, straight line walking test was positive, and there was no abnormality in other neurological examination.

**Figure 1:**
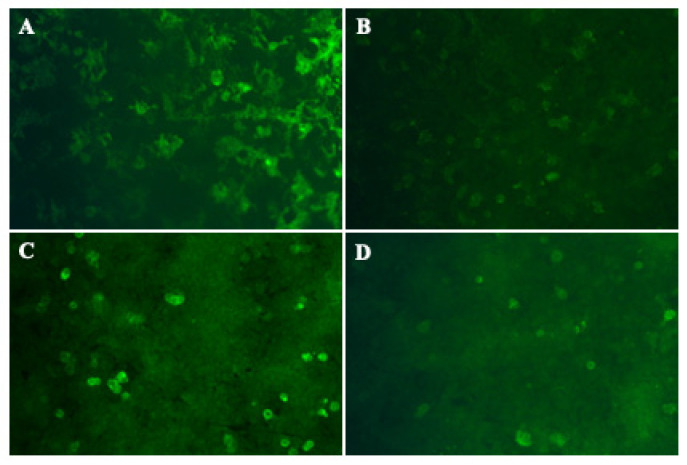
Reactivity to NF 155 and NF186 by CBA Anti-NF 155 IgG4 antibodies detected in the patient’s serum (**A**) (titer 1:1000) and CSF (**B**) (titer 1:100) using CBA method. Anti-NF186 IgG4 antibodies detected in the patient’s serum (**C**) (titer 1:100) and CSF (**D**) (titer 1:100) using CBA method. CBA: cell-based assay; CSF: cerebrospinal fluid; Ig: immunoglobulin; NF: neurofascin.

Laboratory tests showed normal levels of liver function, kidney function, blood glucose, serum electrolytes, blood clotting function, serum creatine phosphokinase, thyroid function, rheumatic immune related antibodies, folate. Serum human immunodeficiency virus antibody, syphilis rapid plasma regain, hepatitis infection, and T-SPOT were negative. Serum tumor markers were negative except for the abnormal cytokeratin 19 and free/total prostate specific antigen ratio. No M-protein was detected in serum and urine. The serum vitamin B12 level > 1500 pg/mL. The cranial, thoracic spine and lumbar spine MRI showed no obvious abnormalities. Cervical MRI revealed degenerative changes. MRI of the right tibia and fibula showed muscle atrophy of the right calf, and the medial muscle group was scattered with patchy abnormal signals. MRI of the left tibia and fibula showed suspected abnormally high signal intensity in the inner calf muscles of the left leg (T2WI). There was no obvious abnormality in the routine electroencephalogram (EEG). Subsequently, lumbar puncture was performed with the CSF pressure was 170 mmH_2_O. Routine CSF analysis revealed that the leukocyte count was 1 × 10^6^/L, Pandy’s test was positive (++), protein was 1773.56 mg/L, chloride was 136 mmol /L, glucose was 3.38 mmol/L, albumin quotient (index) was 40 and IgG oligoclonal bands (OCB) was negative. Then, a series of antibodies of noda-paranodopathy diseases were screened by cell-based assay (CBA) for both serum and CSF. The results showed that the antibodies against NF155 IgG4 (serum 1:1000 and CSF 1:100) and NF186 IgG4 (serum 1:100 and CSF 1:100) were positive for both serum and CSF (Figure 1), while antibodies against CNTN1, CNTN2, CASPR1 and myelin-associated glycoprotein (MAG) were negative. The proportion of CD19+ was 10.4, the proportion of CD20+ was 10. NCV test showed that the detection of nerve distal motor latency (DML) was significantly delayed this time, motor nerve conduction velocity (MCV) was significantly delayed, compound muscle action potential (CMAP) amplitude decreased with different degrees, CMAP of the lower limbs could not be elicited, the CMAP amplitudes of the bilateral proximal ulnar nerve decreased significantly compared with the distal segment, there was conduction block, and the amplitudes of sensory conduction velocity (SCV) and sensory nerve action potential (SNAP) could not be measured. The latency period of F wave of ulnar nerve was prolonged, and the appearance rate was decreased. The detailed features of NCV test were summarized in Table 1. Electrophysiological diagnosis suggested that multiple peripheral neuropathy (involving movement and sensation, mainly demyelination).

**Figure 2:**
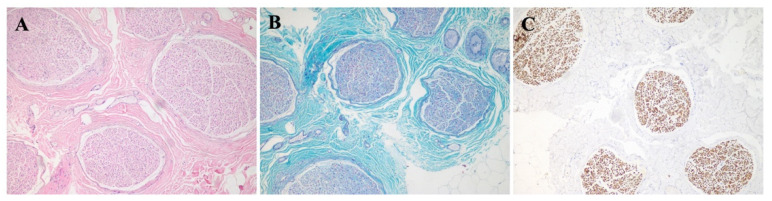
Pathological images of sural nerve biopsy from the patient (**A**) Hematoxylin-eosin (HE) staining of sural nerve biopsy. (**B**) Modified Gomori trichrome (MGT) staining showed no obvious abnormality in the density of myelinated nerve fibers, and no significant demyelination. (**C**) Neurofilament (NF) staining showed that the axonal density was well. (**A**–**C**) Original magnification ×10.

A nerve biopsy of the left sural nerve showed no obvious abnormality in the density of myelinated nerve fibers, and no significant demyelination (Figure 2A–C).

**Figure 3:**
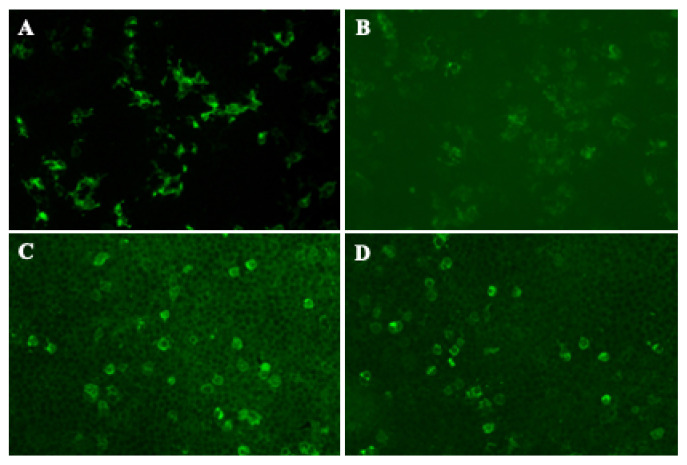
The repeat serum and CSF antibody tests for NF 155 and NF186 by CBA after 4 months follow-up visit. Anti-NF 155 IgG4 antibodies detected in the patient’s serum (**A**) (titer 1:1000) and CSF (**B**) (titer 1:10) using CBA method. Anti-NF186 IgG4 antibodies detected in the patient’s serum (**C**) (titer 1:1000) and CSF (**D**) (titer 1:10) using CBA method. CBA: cell-based assay; CSF: cerebrospinal fluid; Ig: immunoglobulin; NF: neurofascin.

According to the clinical-immune-pathological test results, the patient was diagnosed with anti-NF155/NF186 IgG4 antibody-positive associated with AN, excluded other congenital or acquired peripheral neuropathies. After eliminating the contraindications, he was given RTX therapy (375 mg/m^2^) with improvement of motor and sensory deficit. A follow-up at 4 months demonstrated a marked improvement. He was able to run about 100 m, and said that the distal paraesthesias of lower limbs were much better than before. Repeat serum and CSF antibody tests showed that the antibodies against NF155 IgG4 and NF186 IgG4 in serum were both 1:1000, in CSF were both 1:10 (Figure 3).

## 2. Discussion

Our study describes a case with anti-NF155/NF186 AN that has good response to rituximab (RTX), which are in line with the previous studies [22,23,24]. However, there are still some questions to be clarified. First, the NF186 antibody in this case was weakly positive compared with NF155 antibody, and it might be a nonspecific reaction. Second, why the anti-NF186 antibodies were increased in serum when the patient showed marked improvement? Were the antibodies to NF186 not involved in the pathogenetic mechanism of this case? The possible reasons are as follows: first, the reexamine time is too short; second, after the B cell depletion treatment, it takes time to eliminate the antibodies in the circulation or in the CSF, and the antibody titer needs to be followed up again; third, antibody titers are not fully proportional to clinical symptoms, and the decline of antibody titers is sometimes later than the recovery of clinical symptoms. However, the decrease of CSF titer indicates that the blood-brain barrier may be repaired.

Among anti-NF155 IgG4 antibody patients, histological examinations of the biopsied sural nerves revealed subperineurial edema and occasional paranodal demyelination, but no vasculitis, inflammatory cell infiltrates, or onion bulbs [25]. Even years after the onset of the disease, the loss of myelinated fiber was mild [1]. In anti-NF155 antibody-positive patients but not seronegative AN patients, electron microscopy observed that the terminal Schwann cell loops detached from axons at the paranodes disrupting septate-like transverse bands [3,26,27].

The neuroimaging features of anti-NF186 AN have not yet been clarified. Only one study [19] performed a sural nerve biopsy in a patient with anti-NF140/186 IgG3. It was observed that the microvilli were absence and replaced by elongation of Schwann cell cytoplasm, thereby blocking the node of Ranvier. Degeneration of distal axon was presented. Compared to anti-NF155 patients, axon seemed to be more vulnerable in patients with anti-NF186. Most patients with anti-NF186 IgG were responsive to IVIG treatments and showed a good response to steroids than patients with anti-NF155 IgG4 [9,10,11].

The so-called anti-pan-NF-associated neuropathies caused by the co-existence of anti-NF186/140 and NF155 antibodies were extremely rare and caused life-threatening symptoms such as almost locked-in syndromes [9,11,12,19,20,28]. Burnor et al. [10] reported a 50-year-old man who developed rapidly progressive weakness and paresthesias, which evolved into extraocular weakness, ptosis, facial diplegia, dysarthria, almost complete ophthalmoplegia, quadriplegia, and oscillating sympathetic hypersensitivity with labile blood pressure. Finally, RTX was applied and clinical improvement was achieved around 4 months after onset. Moreover, Vallat et al. [19] described a patient with tetraplegia, respiratory failure, almost locked-in syndrome, and IgG3 autoantibodies against NF186/140 and NF-155. Ultrastructural analysis of nodal architecture revealed occlusion of the nodal gap by myelin layers in the patient. It is worth noting that these patients showed the most severe clinical phenotype and a very long course of disease. Therefore, as also reported in other autoimmune diseases, multiple epitopes may be a predictor of disease severity. Stengel et al. [11] proposed more severe form of anti-pan-NF-associated IgG3 neuropathy, which was similar to Burnor et al.’s cohort, except for IgG subclass autoantibodies. While in Delmont’s study [9], the IgG subclass of anti-NF186/140 was IgG4 in four patients and IgG3 in one patient. Cortese et al. [20] reported a man with a paraparesis, suffered from an anti-pan-NF-associated neuropathy, mainly of IgG4-subtype.

In this case, the symptoms were quite mild and revealed an effective clinical response to IVIG, so no further immunotherapy was required. Fels et al. [28] described a case of fulminant anti-pan-NF-associated neuropathy as IgG4 and IgG3-mediated disease with severe clinical damage. The patient presented with progressive tetraplegia, sensory impairment, cranial nerve involvement, autonomic dysregulation and respiratory insufficiency. The electromyography (EMG) showed abnormal spontaneous activity, including fibrillation and positive sharp waves, accompanied by distal accentuation. Peripheral nerves were not excitable by NCS. Sural nerve biopsy showed active inflammatory neuropathy with mild signs of demyelination.

In summary, the antibody mentioned above is one of the specific biomarkers of AN discovered in recent years. When severe peripheral nerve damage occurs, especially accompanied by tremor, ataxia, and poor response to IVIG treatment, it should be considered that this subtype may be possible, and relevant antibodies should be tested in time. At present, the knowledge and understanding of anti-NF155/NF-186 IgG4 antibody-positive AN is still relatively less in China. So, we strive to achieve early diagnosis and provide more active and targeted treatment and prevention of recurrence and aggravation as soon as possible.

## Figures and Tables

**Table 1:** The nerve conduction velocity (NCV) features of the patient.

**MCV**	**Latency (ms)**	**Amplitude (mV)**	**Distance (mm)**	**Conduction** **Velocity (m/s)**	**Average F-M Latency (ms)**
Ulnar nerve (left)					
Wrist-ADM	5.48	1.46			51.1
Below elbow-wrist	12.8	0.54	210	28.7	
Above elbow-below elbow	16.5	0.73	90.0	24.3	
Ulnar nerve (right)					
Wrist-ADM	5.12	2.0			56.8
Below elbow-wrist	15.5	0.96	220	21.2	
Above elbow-below elbow	18.1	1.00	80.0	30.8	
Median nerve (left)					
Wrist-APB	6.46	3.3			
Elbow-wrist	16.2	2.9	270	27.7	
Median nerve (right)					
Wrist-APB	7.13	5.0			
Elbow-wrist	16.0	3.7	278	31.3	
Tibial nerve (right)					
Ankle-AH	--	--			--
Common peroneal nerve (left)					
Ankle-EDB	--	--			
Common peroneal nerve (right)					
Ankle-EDB	--	--			
Common peroneal nerve (anterior tibialis) (right)					
Fibular head-anterior tibialis	--	--			
**SCV**	**Latency (ms)**	**Amplitude (uV)**	**Distance (mm)**	**Conduction Velocity (m/s)**	
Ulnar nerve (left)					
Finger V-wrist	--	--			
Ulnar nerve (right)					
Finger V-wrist	--	--			
Median nerve (left)					
Finger II-wrist	--	--			
Median nerve (right)					
Finger II-wrist	--	--			
Superficial peroneal nerve (left)					
Ankle-dorsum pedis	--	--			
Superficial peroneal nerve (right)					
Ankle-dorsum pedis	--	--			

ADM, abductor digiti minimi; AH, abductor hallucis; APB, abductor pollicis brevis; EDB, extensor digitorum brevis; MCV, motor conduction velocity; SCV, sensory conduction velocity.

## Data Availability

The data generated for this study are available on request to the corresponding author.
